# A22 INVESTIGATING THE EFFECTS OF DIET TRIGGER DISCLOSURE ON BEHAVIOUR IN IRRITABLE BOWEL SYNDROME (IBS) PATIENTS WITH PERCEIVED GLUTEN SENSITIVITY

**DOI:** 10.1093/jcag/gwad061.022

**Published:** 2024-02-14

**Authors:** C Seiler, G H Rueda, P Miranda, A Nardelli, R Borojevic, A Hann, C Southward, S Rahmani, R De Souza, A Caminero, D Schuppan, P Moayyedi, E Verdu, S Collins, M Pinto-Sanchez, P Bercik

**Affiliations:** Medicine, McMaster University, Hamilton, ON, Canada; Medicine, McMaster University, Hamilton, ON, Canada; Medicine, McMaster University, Hamilton, ON, Canada; Medicine, McMaster University, Hamilton, ON, Canada; Medicine, McMaster University, Hamilton, ON, Canada; McMaster University, Hamilton, ON, Canada; Medicine, McMaster University, Hamilton, ON, Canada; Medicine, McMaster University, Hamilton, ON, Canada; Medicine, McMaster University, Hamilton, ON, Canada; Medicine, McMaster University, Hamilton, ON, Canada; Johannes Gutenberg Universitat Mainz, Mainz, Rheinland-Pfalz, Germany; Medicine, McMaster University, Hamilton, ON, Canada; Medicine, McMaster University, Hamilton, ON, Canada; Medicine, McMaster University, Hamilton, ON, Canada; Medicine, McMaster University, Hamilton, ON, Canada; Medicine, McMaster University, Hamilton, ON, Canada

## Abstract

**Background:**

Patients with self-perceived gluten sensitivity often undergo double-blinded, placebo-controlled (DBPC) challenge studies to determine whether gluten or wheat trigger their symptoms. However, it is unknown whether the result disclosure impacts patients’ beliefs and dietary choices.

**Aims:**

To evaluate the impact of disclosing results of DBPC challenge with gluten and wheat on beliefs and dietary choices in IBS patients who adopt a gluten-free diet (GFD).

**Methods:**

We conducted a DBPC crossover trial in 28 adult IBS patients (Rome IV) who previously reported improvement of symptoms while on a GFD. Patients were on a GFD throughout the study and were challenged for 7 days with whole wheat, purified gluten, and nocebo (gluten-free flour) added to low-FODMAP cereal bars, followed by 2-week washouts. Genetic predisposition to celiac disease and anti-gliadin IgG (AGA) were assessed. At least 6 months after the study completion, patients were contacted to assess their current diet, dietary beliefs, and symptoms prior to disclosing their study results. We assessed the same outcomes one month later. Statistical comparisons used paired Wilcoxon signed rank tests.

**Results:**

The DBPC study showed similar proportions of participants reacting to wheat, gluten, and nocebo challenge, with greater symptoms after wheat compared to baseline (P<0.05). AGA IgG were present in 8% (2/25) and celiac disease-related genes in 80% (20/25) of patients. Out of 28 participants, 26 completed the pre-disclosure and 25 completed post-disclosure follow-up. All participants (N=26) believed that at least one challenge triggered their symptoms: whole wheat N=22, pure gluten N=23, nocebo N=2. Prior to disclosure, 17 participants continued while 9 abandoned the GFD; none changed their diet post-disclosure. Reasons to stay on the GFD included belief in its efficacy (N=16) and better quality of life (N=15), while reasons for abandoning included difficulty to adhere to the GFD and its cost (N=6) and losing belief in its efficacy (N=3). IBS symptoms did not change pre- vs. post-disclosure. Participants not reacting to wheat or gluten challenge expressed slightly lower median belief in GFD efficacy pre- vs. post-disclosure (80%, IQR [64%, 97%] vs 71%, IQR [48%, 78%]; P=0.03; Figure 1). Those worsening after wheat or gluten did not change their beliefs.

**Conclusions:**

Although most IBS patients with self-perceived gluten sensitivity had celiac disease predisposition, only some reacted to gluten or wheat. Furthermore, after disclosing the study results, all participants maintained their dietary habits avoiding gluten, thus suggestive of strong central mechanisms underlying their symptoms.

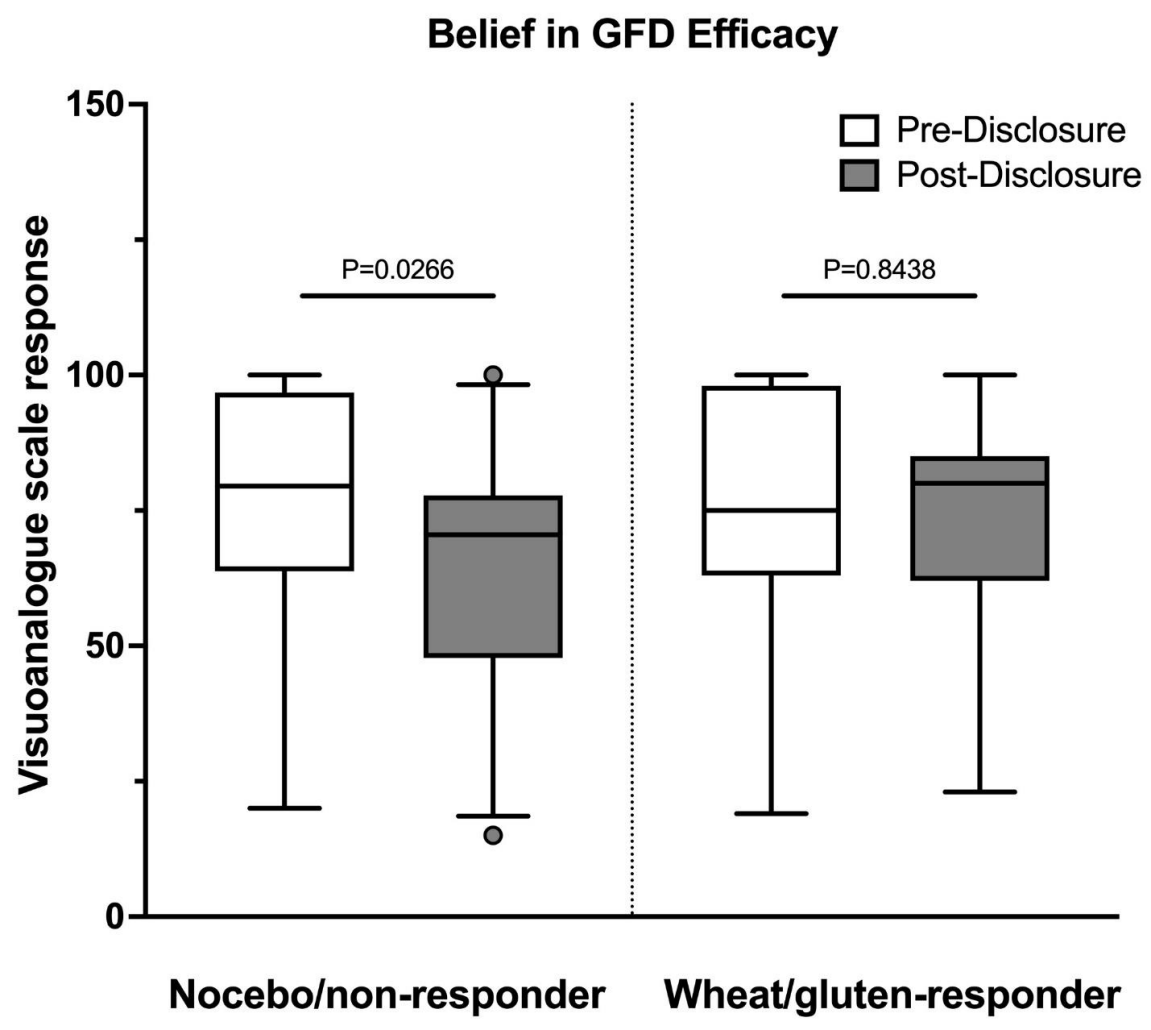

Figure 1. Belief in GFD efficacy pre- vs. post-disclosure of study results.

**Funding Agencies:**

CIHRSociety for the Study of Celiac Disease, Nestle

